# A criterion for assessing obstacle-induced environmental complexity in multi-robot coverage exploration

**DOI:** 10.1371/journal.pone.0323112

**Published:** 2025-05-16

**Authors:** Khalil Al-rahman Youssefi Darmian, Reza Abbaszadeh Darban, Gregor Kastner, Wilfried Elmenreich

**Affiliations:** 1 Institute of Networked and Embedded Systems, University of Klagenfurt, Klagenfurt am Wörthersee, Carinthia, Austria; 2 Department of Computer Engineering, Ferdowsi University of Mashhad, Mashhad, Razavi Khorasan, Iran; 3 Department of Electrical Engineering and Computer Science, Lassonde School of Engineering, York University, Toronto, Ontario, Canada; 4 Department of Statistics, University of Klagenfurt, Klagenfurt am Wörthersee, Carinthia, Austria; Beijing Institute of Technology, CHINA

## Abstract

In many applications, such as coverage exploration and search and rescue missions, accurately assessing environmental complexity is valuable for performance evaluation and algorithm adjustments. Despite this, in the context of multi-robot systems, quantifying environmental complexity caused by obstacles when using autonomous ground robots presents significant challenges. This research proposes a criterion for measuring environments’ obstacle-induced complexity in the context of autonomous multi-robot coverage exploration. The criterion rates the environment’s complexity numerically, where 0 denotes obstacle-free setups, and the value increases with obstacle-related effects, reaching a maximum of 1, representing the highest measurable complexity for the criterion. The proposed criterion is independent of robot hardware specifications and algorithm-specific aspects. Furthermore, it is independent of the environment’s size and the ratio of the area occupied by obstacles, enabling comparisons across various environments. Statistical analysis shows the metric performs well both on average and in single-case comparisons.

## Introduction

In the realm of robotics, regardless of the specific tasks or functions robots are designed to perform, their performance is inherently influenced by the environments in which they operate. This influence varies significantly depending on the nature of the environment and the task. For instance, environmental factors such as wind can greatly affect the performance of drones operating in desert regions, while obstacles pose challenges to robots navigating on the ground.

In certain applications, it is possible to minimize the environmental impact on robots by constraining their Degrees of Freedom (DoF) [1] or limiting their range of motion. This approach can be seen in systems designed for task-specific operations, such as industrial robotic arms [2], 3D printers [3], pick-and-place robots [4], and CNC machines [5]. By carefully adjusting the design and operational constraints, these robots can perform tasks with minimal interference from environmental factors [6].

However, in other tasks, such as those involving warehouse robots [7,8], search and rescue robots [9–11], robotic cleaners [12,13], and, more broadly, mobile robotic systems, the ability to mitigate environmental effects is limited. For instance, coverage exploration tasks require robots to traverse and explore entire environments, making it impractical to constrain their operational boundaries. In such scenarios, quantifying the complexity of the environment becomes essential. Understanding this complexity is crucial to have a fair evaluation of different algorithms and compare their performance more effectively [14]. Furthermore, in some planning and decision-making, such as predicting shortest path costs [15] and decision-making in dynamic environments [16], prior knowledge of environmental complexity can facilitate a better understanding of potential outcomes and the estimation of optimal results.

Determining environmental complexity is particularly challenging in the context of multi-robot coverage exploration. The simultaneous presence of multiple robots increases the likelihood of encountering obstacles and influences the coverage process. Conversely, multi-robot coverage systems offer advantages such as scalability, enabling adaptation to environments of varying sizes and complexities.

Measuring environmental complexity in multi-robot systems can also aid in determining the optimal number of robots required for a given task. Specifically, it may help identify the point at which increasing the number of robots no longer reduces the computed environmental complexity. This represents a golden team size, beyond which additional robots would not provide further benefits in terms of complexity reduction. This optimization can save a substantial cost in industry by minimizing the number of robots required for efficient task completion. However, the mentioned golden team size may not hold in specific problems under extreme conditions, where one objective takes priority over others (e.g., in search and rescue missions) or where cost functions impose hard limits (e.g., no gain if the problem is not solved within a critical time frame).

This paper presents a quantitative criterion for assessing environmental complexity from the perspective of multi-robot systems, specifically in the context of the coverage exploration problem, which involves multiple robots. In this context, environmental complexity is defined as the effect of obstacle structures on the robots’ coverage exploration tasks. The introduced criterion calculates how the structure of obstacles in the environment influences the robots’ activities, quantifying the extent to which environmental obstacles impact their tasks. The criterion produces a numerical value ranging from 0, indicating an obstacle-free environment (essentially a reference environment), to 1, indicating an infinitely complex environment. Notably, in some cases, the environment may assist the robots’ objectives, leading the criterion to yield a negative complexity value. For example, in coverage exploration tasks, certain obstacle configurations can simplify the coverage process by limiting the decision space of robots compared to an obstacle-free environment of the same size, resulting in a negative complexity measurement.

The proposed criterion is entirely independent of the robots’ physical attributes, their inter-communication, error rates in their actions within the environment, or the specific features of their guiding algorithms. Furthermore, it remains unaffected by the environment’s size or the percentage of the area covered by obstacles. This independence enables the comparison of various environments across different scenarios and applications.

The results demonstrated that the proposed criterion effectively assesses environmental complexity in multi-robot systems, providing a reliable way to compare different environments. It remains unaffected by factors like geometric shape, size, and obstacle coverage, making it versatile across various scenarios. The analysis revealed that in more complex environments, adding more robots can reduce the perceived complexity, while in simpler environments, the number of robots has little impact. Thus, the criterion reflects both the advantages and disadvantages of multi-robot systems, capturing their influence on environmental complexity. Furthermore, a statistical analysis was carried out to suggest different estimators, such as point estimators like the mean and median for on-average comparisons and distribution-based comparisons for single-run assessments.

The structure of this paper proceeds as follows: the next section provides an overview of previous research on quantitative assessments of environmental complexity in robotics applications. Next, the methodology section describes the proposed criterion. After that, the results section delves into an analysis of the outputs generated by the proposed metric. Finally, the conclusion section summarizes the paper’s findings and offers insights into potential future research directions.

## State of the art

Numerous studies have explored the influence of environmental complexity on robotic systems. This section provides an in-depth review of this field’s most relevant and recent research.

Ermacora *et al*. [17] introduced a framework to assess the effect of environmental complexity on the performance of autonomous robots. The methodology adopted in this approach consists of two primary stages: firstly, it involves a comparison between the actual navigation performance of robots and their optimal performance, which is computed within the same environmental context. Secondly, these performance evaluations are then linked to the complexity of the environment. In this research, a metric named Mean Shortest Path is introduced to measure the environmental complexity, relying on the average shortest path distance between any two physically accessible points in the environment. Ho *et al*. [18] also introduced a framework for comparing robotic tasks motivated by the impact of task complexity on robot performance. The task includes what robots must accomplish and the environment in which operations must be performed.

Evaluating the impact of the environment on the performance of robot sensors and the accuracy and quality of the data they can obtain is another method for assessing environmental complexity. For example, in the context of computational-sensory systems, Donald [18,19] suggests that when two sensors possess equivalent capabilities, they likely perceive the same level of environmental complexity. However, if one sensor is considerably more powerful than the other, assumptions about their perception of environmental complexity become less certain.

Another method for measuring environmental complexity is to measure the disruption caused by obstacle structures in the movement of robots. Shell and Mataric [14] introduced a concept known as space syntax [20]. The space syntax theory, as opposed to emphasizing distance, primarily focuses on the connectivity of spatial features when analyzing spatial structure [21]. This scale-invariant perspective contributes to its widespread applicability; in this research, it refers to normal human movement within structured space and crowd evacuation scenarios.

Furthermore, Anderson and Yang [22] demonstrate that two factors, namely entropy and compressibility of the environment, determine its perceived complexity. In this research, complexity is influenced by the amount of open space in the environment, as when a robot encounters a zone of free space, it faces the challenge of selecting the optimal path. Making an incorrect decision can lead to increased travel time to reach its intended destination. Assuming the environment is represented as a binary matrix (0 for obstacles and 1 for open areas), the entropy of this grid is used to measure the complexity of the environment. Also, similar obstacle structures in an environment reduce the chance of using them as landmarks and increase environmental complexity. Thus, this research counts compressibility (i.e., the possibility of compressing environment binary matrix) as another complexity measure. In another research [23], authors claimed that their complexity measure could predict the average number of steps for a robot searching task with 90% accuracy. However, only a single experiment is reported. Additionally, Sartori *et al*. [24] employed the two metrics introduced in [22] and [17] to train a Convolutional Neural Network (CNN). The CNN takes an image of the target environment as input and produces the corresponding values of the two metrics as output. This research aims to expedite the process of calculating environmental complexity.

Although various methods for evaluating environmental complexity are available, they generally concentrate on structural properties without accounting for the effects of team size and the specific tasks the robotic team aims to accomplish. The proposed method not only assesses the influence of obstacles on efficiency but also considers how team size can counter the complexities introduced by obstacles and the characteristics of coverage tasks that increase the likelihood of encountering these obstacles. To the best of the authors’ knowledge, no existing approach incorporates these considerations, which complicates direct comparisons.

## Methodology

The underlying assumption is that the complexity of an environment heavily influences robotic operations and the performance of multi-robot systems. However, as the number of robots increases, to a certain extent, the environmental complexity impact on performance diminishes, as multiple robots can encounter obstacles in parallel, reducing the overall effect on the system. The proposed criterion measures this effect by comparing the performance of a multi-robot system in the presence of obstacles (target environment) to its performance in the absence of obstacles (base environment), with the same number of robots in both environments. Additionally, this test can be performed for the same environment using different numbers of robots, which would yield different results.

In this paper, the coverage exploration problem, i.e., the task in which multiple robots aim to visit all regions of an environment, is specifically considered, and the results are valid only for this application. The proposed criterion may be applicable to other applications, such as swarm robotic search in complex unknown environments, though further validation is required. However, in all comparisons, it is essential to maintain the conditions that critically influence the system’s effectiveness. For instance, in the coverage exploration problem discussed here, it is assumed that the robots are initially scattered randomly throughout the environment. Alternatively, one could consider scenarios where all robots begin the exploration task from a single starting point, such as a corner of the environment.

Since the proposed criterion relies on statistical interpretations, multiple simulation runs are required to reliably evaluate the effects of target environments on multi-robot systems within the desired application. A basic algorithm that is computationally efficient and capable of performing the required task must be employed to ensure feasibility.

To this end, the Greedy Coverage Algorithm (GCA) was used. In GCA, robots iteratively aim to move toward the nearest least visited cells, denoted as $C_{\text{least}}$, to maximize exploration coverage. Robots maintain the number of times each cell is visited by the whole swarm to guide their movement decisions. Each robot determines its next destination by locally evaluating the visit counts of cells in the immediate vicinity. This approach is simple and computationally inexpensive. The steps of the GCA are outlined in Algorithm 1. In this algorithm, $E^{*}$ is the set of all cells on the grid, ℰ is the set of all cells on the grid not occupied by an obstacle or other robots, V(c) is the number of visits to cell $c\in E^{*}$, ℛ is the set of all robots, and *p*_*i*_ is the position of robot *i*.

**Algorithm 1. Greedy Coverage Algorithm (GCA) for a Multi-Robot System.**

1: Initialize visit count $V(c)=0\;\forall c\in E^{*}$

2: Initialize visit count $V(c)=1\;\forall c\in E^{*}$ that is occupied by

an obstacle.

3: **while** there exist unvisited cells in $E^{*}$ **do**

4:   **for all** robot $r_{i}\in ℛ$ **do**

5:    Identify neighboring cells ${𝒩}_{i}=\{c\in ℰ|c$ is adjacent to

*p*_*i*_}

6:    Select the least visited cells $C_{\text{least}}=\{c\in {𝒩}_{i}|V(c)=$

$\min_{c^{\prime }\in {𝒩}_{i}}V(c^{\prime })\}$

7:     **if** $|C_{\text{least}}|>1$ **then**

8:      Select a cell c^*^ randomly from $C_{\text{least}}$

9:     **else if** $|C_{\text{least}}|=1$ **then**

10:      $c^{*}=C_{\text{least}}[1]$

11:     **else**

12:      $c^{*}=p_{i}$

13       **end if**

14:     **if** $c^{*}\neq p_{i}$ **then**

15:      Move robot *r*_*i*_ to c^*^

16:     **end if**

17:     Increment visit count $V(c^{*})\leftarrow V(c^{*})+1$

18:   **end for**

19: **end while**

To measure the *Complexity* of the target environment, the time for a *Complete Traversal (CT)* of the Target Environment (${\text{CT}}_{\text{TE}}$) by the multi-robot system must be compared to the case of the Obstacle-free Environment (${\text{CT}}_{\text{OfE}}$) of the same size and with the same number of robots. *CT* is defined as the total time taken by the system to complete the coverage exploration task, starting from the first robot’s movement until the final robot visits the last unvisited cell in the environment. Eq 1 shows the proposed relation of the introduced terms. As it shows, *Complexity* is expected to increase as ${\text{CT}}_{\text{TE}}$ increases and decreases as ${\text{CT}}_{\text{OfE}}$ goes close to ${\text{CT}}_{\text{TE}}$. Moreover, the effect of the size of the environment is embedded in the coverage time, and since the division cancels out the time unit, the *Complexity* is unitless and independent of the size of the environment.

$$\text{Complexity}\propto \frac{{\text{CT}}_{\text{TE}}}{{\text{CT}}_{\text{OfE}}}$$ (1)

However, because the effect of obstacles on the system’s performance may be significant, ${\text{CT}}_{\text{TE}}$ may become large, and thus *Complexity* may increase significantly. To keep *Complexity* within a limited range, ${\text{CT}}_{\text{TE}}$ and ${\text{CT}}_{\text{OfE}}$ must swap places (Eq 2). This way, as ${\text{CT}}_{\text{TE}}\to +\infty$, $\text{Complexity}\to 0^{-}$. On the other hand, as ${\text{CT}}_{\text{TE}}\to {\text{CT}}_{\text{OfE}}$, Complexity→−1. In Eq 2, the negative sign is necessary to correct the change direction of the *Complexity*.

$$\text{Complexity}\propto -\frac{{\text{CT}}_{\text{OfE}}}{{\text{CT}}_{\text{TE}}}$$ (2)

To remove the negative sign of the *Complexity* and resolve its reverse changing direction, Eq 2 must be updated to Eq 3. In the new formula, *Complexity* goes to 1^−^ when ${\text{CT}}_{\text{TE}}$ goes to infinity (Eq 4), and *Complexity* of the base environment (i.e., ${\text{CT}}_{\text{TE}}={\text{CT}}_{\text{OfE}}$) is 0.

$$\text{Complexity}\propto 1-\frac{{\text{CT}}_{\text{OfE}}}{{\text{CT}}_{\text{TE}}}$$ (3)

$$\lim_{{\text{CT}}_{\text{TE}}\to \infty }\text{Complexity}=1^{-}$$ (4)

Another influencing factor in calculating the *Complexity* is the Traversable Area (TA) in an environment, which is equal to the total area of the environment minus the area occupied by obstacles. Eq 5 shows the formula for calculating a normalized TA, denoted as ${\text{TA}}^{\prime }$. According to this equation and based on the definition of the Obstacle-free Environment, ${\text{TA}}_{\text{OfE}}^{\prime }=1$, while ${\text{TA}}_{\text{TE}}^{\prime }\in (0,1]$.

$${\text{TA}}^{\prime }=\frac{\text{environment size}-\text{obstacle-occupied area}}{\text{environment size}}$$ (5)

By applying these factors as normalizing coefficients for *CT* times, the effect of the area occupied by obstacles is also negated (Eq 6).

$$\text{Complexity}=1-\frac{\frac{{\text{CT}}_{\text{OfE}}}{{\text{TA}}_{\text{OfE}}^{\prime }}}{\frac{{\text{CT}}_{\text{TE}}}{{\text{TA}}_{\text{TE}}^{\prime }}}=1-\frac{\frac{{\text{CT}}_{\text{OfE}}}{1}}{\frac{{\text{CT}}_{\text{TE}}}{{\text{TA}}_{\text{TE}}^{\prime }}}=1-\frac{{\text{TA}}_{\text{TE}}^{\prime }\cdot {\text{CT}}_{\text{OfE}}}{{\text{CT}}_{\text{TE}}}$$ (6)

**Fig 1:**
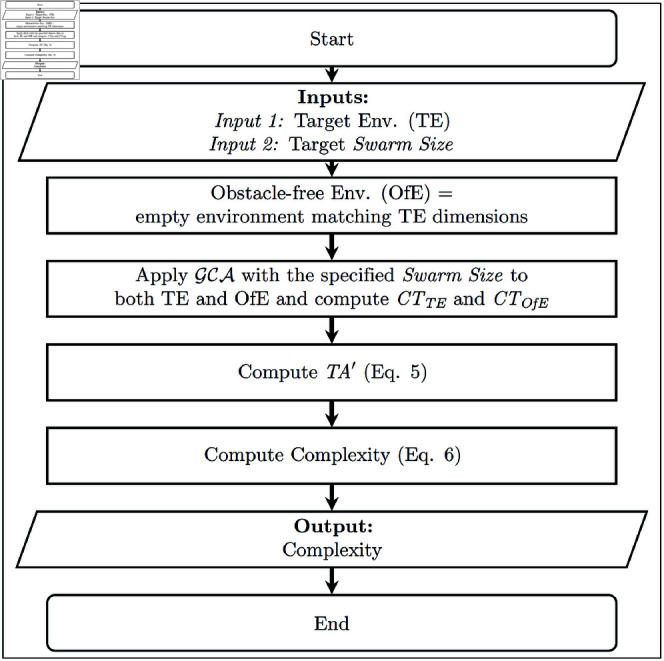
Flowchart of computing the Complexity of a given Target Environment (TE) for a specified Swarm Size.

The resulting complexity criterion is designed to measure the effect of environmental complexity on the performance of multi-robot coverage exploration and the number of robots involved. Fig 1 illustrates the process of utilizing the proposed method to compute the complexity of a given Target Environment (*TE*) for a specified *Swarm Size*. The following section demonstrates the output of the proposed metric for various environments ranging from simple to complex.

## Results

**Fig 2:**
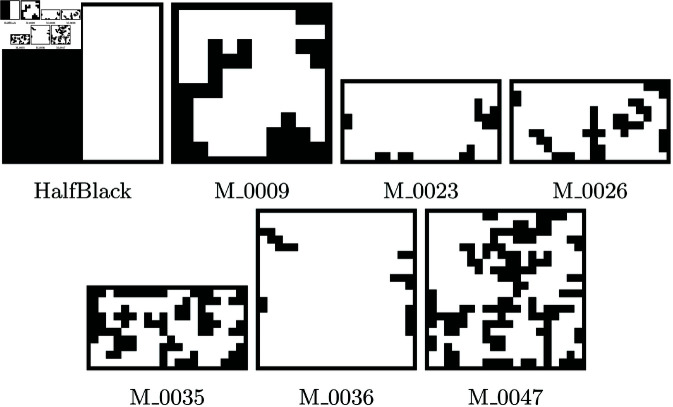
Environments with varying sizes, obstacle occupancy, and complexity levels for evaluating the proposed criterion.

This section focuses on evaluating the performance of the proposed complexity criterion by testing it on different environments, challenging its quality and practicality in comparing environments. Considering that the geometric shape of the environment (e.g., square or rectangular), its size, and the obstacle-occupied area do not directly affect the metric results, the test environments depicted in Fig 2 are selected to have diverse features. These environments are of square and rectangular shapes and comprise simple and complex maps with varying obstacle-occupied areas, creating different scenarios. Table 1 presents information on their size and obstacle-occupied areas.

**Table 1:** Brief data of the seven environments used for experiments.

Environment	Size (units^2^)	Obstacle Occupancy (%)
HalfBlack	200	50
M_0009	73	27
M_0023	183	9
M_0026	160	20
M_0035	120	40
M_0036	381	5
M_0047	268	33

As point estimates for complexity, Table 2 illustrates the mean and median of complexities calculated for each environment utilizing the introduced criterion. To compute the complexity of each environment, 300 simulations with six robots were conducted. Multiple simulations are required to account for the impact of robots’ random decisions on the results. However, relying solely on point estimates of complexity as a means of comparing environments is not sufficiently reliable. Various statistical methods for comparing results in different applications are introduced later in this section. Nonetheless, it is evident that the introduced complexities reflect the relative simplicity of the environments. For instance, environment M_0009 is simpler than environment M_0047. The average complexities calculated for these two environments are 0.27 and 0.66, respectively, confirming that environment M_0009 is much simpler.

**Table 2:** The mean and median of complexities calculated for each environment utilizing the introduced criterion.

Environment	Mean Complexity	Median Complexity
HalfBlack	-0.0643	-0.0363
M_0009	0.2670	0.3004
M_0023	0.0961	0.1240
M_0026	0.3451	0.3554
M_0035	0.7116	0.7276
M_0036	0.0726	0.0834
M_0047	0.6642	0.6709

Moreover, as shown in the table, the estimated complexity of the HalfBlack environment is –0.064, less than the complexity of an obstacle-free environment, which is 0. This is because the obstacle is entirely blocking a large portion of the environment. Thus, the environment’s difficulty is reduced even compared to an obstacle-free environment of the same size.

**Fig 3:**
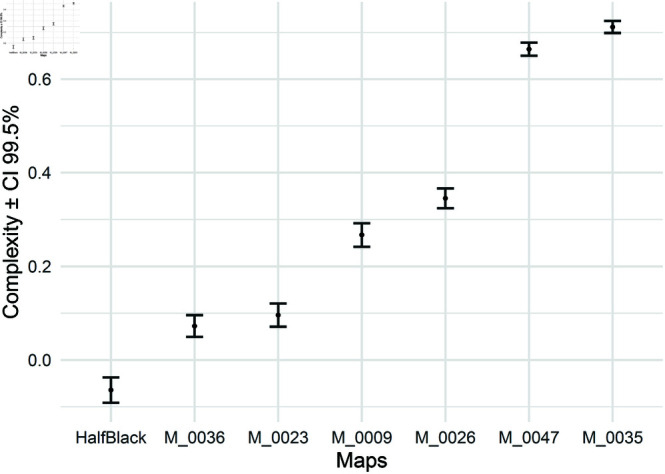
Average and 99% CI (Eq 7) of environmental complexity for maps with a robot count of 6.

As the calculated complexities are influenced by the robots’ random decisions, using point estimates to compare two environments when their complexities are very close is unreliable. Therefore, to address this ambiguity, we also provide confidence intervals (CIs). Fig 3 displays the average complexities calculated for the environments in Fig 2, along with the respective 99% CIs computed using Eq 7. In this equation, *X* represents the computed complexity using the procedure shown in Fig 1, $\bar{X}=\frac{1}{N}\sum_{i=1}^{N}X_{i}$ is the sample mean, *N* is the number of independent simulation runs, and $S=\sqrt{\frac{1}{N-1}\sum_{i=1}^{N}(X_{i}-\bar{X}{)}^{2}}$ is the sample standard deviation. Finally, the value 2.576 is the Z-score for a 99% confidence level obtained from the standard normal distribution.

$$\text{99\textbackslash{}\% CI}=\bar{X}\pm 2.576\times \left(\frac{S}{\sqrt{N}}\right)$$ (7)

If the confidence intervals of two environments do not overlap, these two environments can be considered statistically significantly different on average. For example, environments M_0023 and M_0036 show an overlap in their confidence intervals, indicating that, on average, they are not significantly different. In contrast, for environments M_0009 and M_0023, no overlap is observed in their confidence intervals, implying that these two environments are different on average. It is clear that due to the greater average complexity of environment M_0009, this environment is more challenging than M_0023.

**Fig 4:**
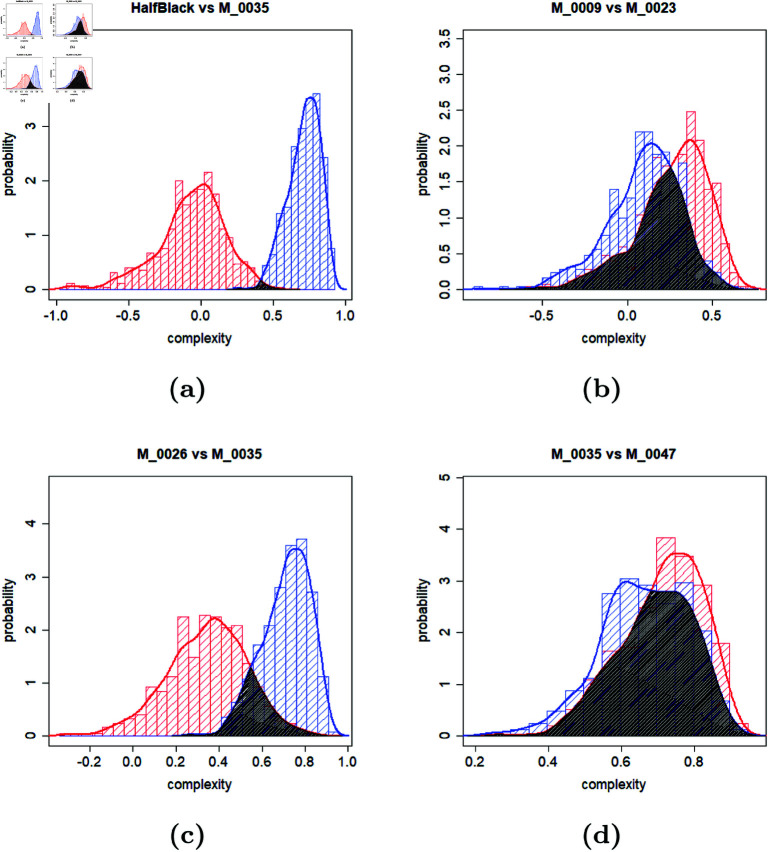
Comparing the distribution of the computed complexities for different environments (maps).

Using point estimates allows for a general comparison of environments. In this way, it can be inferred that, for example, when a mission is executed numerous times in environments M_0009 and M_0023, environment M_0009 is generally more challenging. However, this is not always the practical case. In many real-world applications, only a single or few runs in each environment will be required. In other words, in the previous example, the comparison should answer the question of how confident it can be that robots will face more difficulty in M_0009 compared to M_0023 when only one run is conducted in each environment. A statistical solution to compare environments with a specified confidence level involves comparing the distributions of the calculated complexities to provide a density estimate. Fig 4 illustrates examples of such comparisons.

In Fig 4, the percentage of non-overlapping areas of the distributions reflects the confidence of claiming that the complexity of two environments is distinguishable. Intuitively, comparing two non-overlapping distributions seems straightforward. However, when two distributions overlap, that will be the source of error in the estimation of the more complex environment in a single execution. This stems from the fact that the overlapping area includes observations where the distribution with a lower mean appeared more complex than the one with a higher mean, and vice versa.

**Fig 5:**
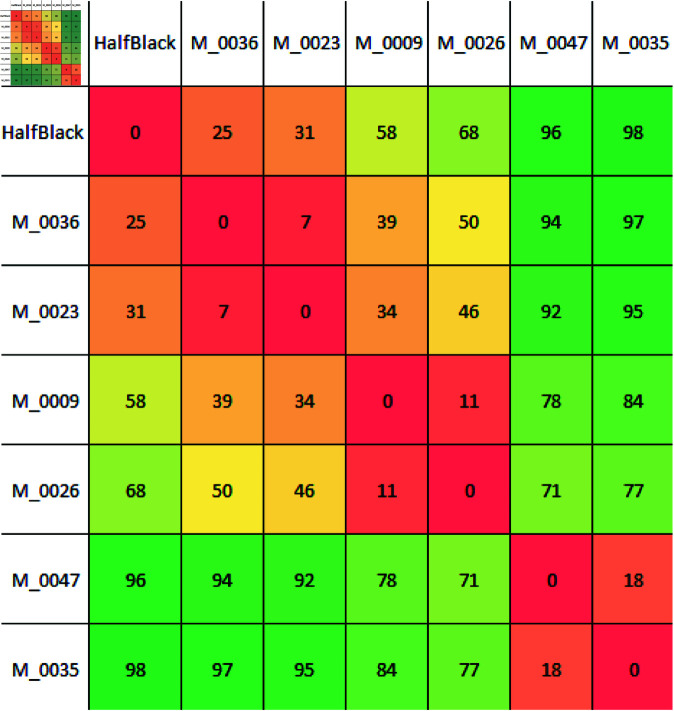
Grid shows the confidence (%) of the proposed criterion on deciding how different each pair of environments is.

Fig 5 presents a matrix of confidences for pairwise environments’ comparisons. In the previous example, considering environments M_0009 and M_0026, although the averages and confidence intervals indicate that these two environments are different, the significant overlap in the distributions with a confidence percentage of only 34% suggests that this difference is not valid for a small number of runs. In contrast, for environments HalfBlack and M_0035, since the overlapping area is tiny, it can be stated that even for a minimal number of runs, M_0035 is more complex than environment HalfBlack.

**Fig 6:**
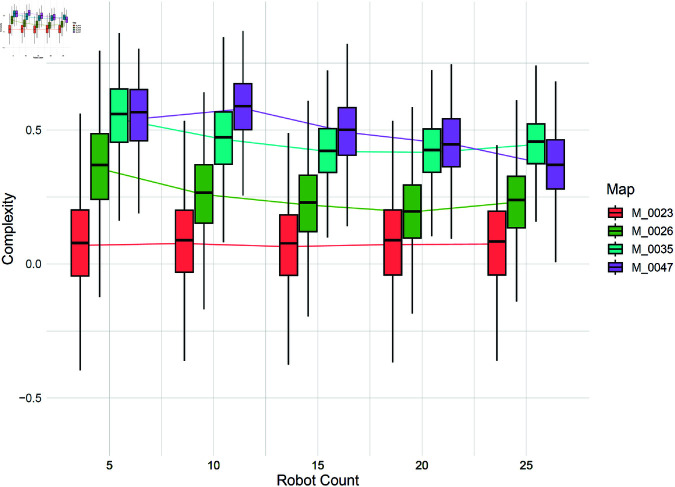
The effect of robot counts on the computed complexity for different environments.

Up to this point, comparisons have been made with a fixed number of 6 robots. However, the main feature of the proposed criterion is its applicability to multi-robot systems. Fig 6 demonstrates the effect of the number of robots on the calculated complexity for four environments. A limited number of environments are shown in this figure to preserve readability. As shown in the figure, the effect of the number of robots on a very simple environment like M_0023 (with an average complexity of 0.1 for 6 robots) is negligible because the environment is inherently simple, and increasing the number of robots does not change its difficulty for the multi-robot system. However, for a complex environment like M_0047 (with an average complexity of 0.66 for 6 robots), increasing the number of robots reduces the difficulty from the perspective of large multi-robot systems, thereby lowering the measured complexity. As another instance, for M_0035 the complexity decreases to its lowest level after reaching a specific threshold of 15 robots. This threshold represents the optimal number of robots for operating in this environments. Beyond this point, increasing the number of robots does not reduce complexity. In some cases, like M_0026, having too many robots may even make the environment appear more complex. This could be an indication of interference in the robots’ activity due to high density.

**Fig 7:**
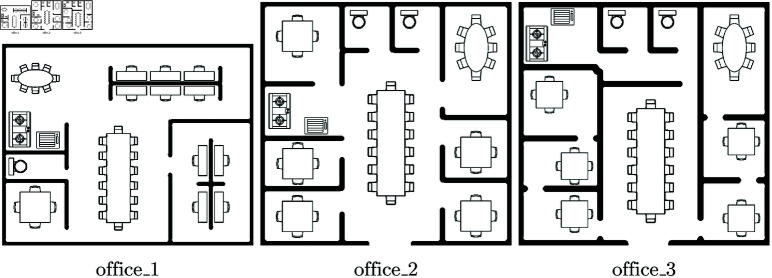
Office floor plans introduced as additional evaluation maps to incorporate realistic spatial constraints, complementing the randomly generated obstacle structures in Fig 7.

To complement the randomly generated obstacle structures used as benchmarks in Fig 2, three office floor plans, illustrated in Fig 7, are introduced to provide a more realistic setting for evaluation. These maps incorporate structured layouts with rooms, corridors, and furniture arrangements that resemble real-world office spaces. Their inclusion allows the proposed method to be assessed under conditions that better reflect practical deployment scenarios, where navigation and coverage are influenced by realistic spatial constraints.

**Fig 8:**
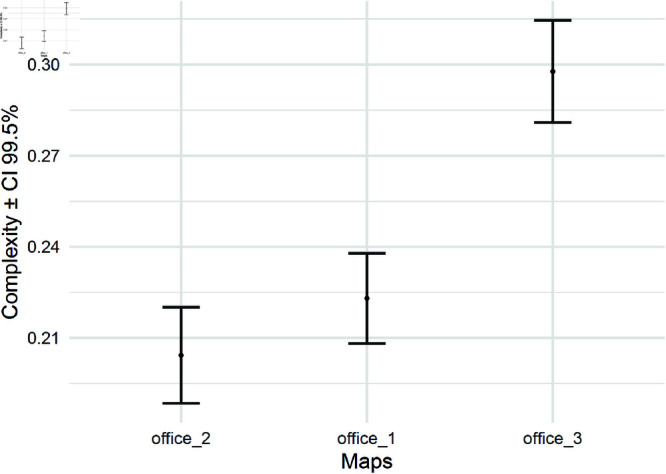
Average and 99% CI (Eq 7) of environmental complexity for maps with a robot count of 6.

Fig 8 presents the computed complexities for the office floor plans shown in Fig 7 using 6 robots. The complexities are displayed with 99% confidence intervals (CIs). The results indicate that the CIs for offices 1 and 2 overlap, suggesting that, based on the proposed criterion, their complexities are not statistically distinguishable on average for this robot count. In contrast, office 3 exhibits a complexity value outside these overlapping intervals, indicating a statistically significant difference in complexity according to the proposed measure. This suggests that while the first two office layouts may present similar navigational and coverage challenges for the multi-robot system, office 3 introduces structural characteristics that result in a noticeably different complexity score at this scale.

**Fig 9:**
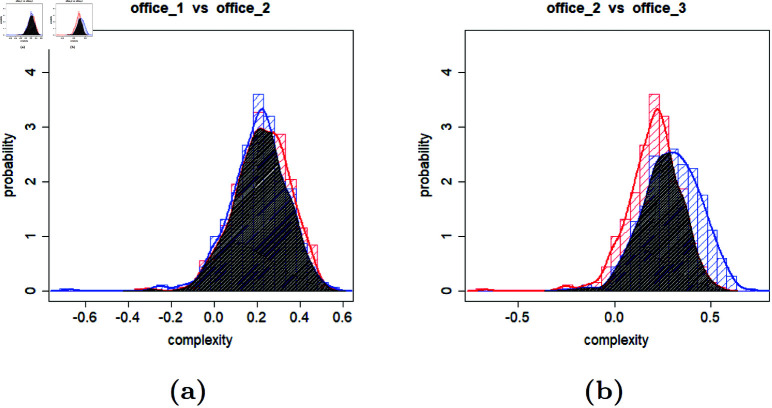
Comparison of computed complexity distributions for office floor plans shown in Fig 7.

Fig 9 compares the distributions of computed complexities between office floor plans. The left plot compares offices 1 and 2, showing an overlapping area of 90%, while the right plot compares offices 2 and 3, with an overlapping area of 70%. While 70% is lower than 90%, indicating a greater distinction between the two distributions, it still implies that for one or a few runs, the complexities of offices 2 and 3 are 70% likely to be similar. This, however, does not contrast with the conclusion from the previous judgments derived from comparing CIs, indicating a statistically significant difference between these two office floor plans on average (i.e., for several runs), but demonstrates how point estimates and distributional comparisons offer complementary perspectives in analyzing environmental complexity.

**Fig 10:**
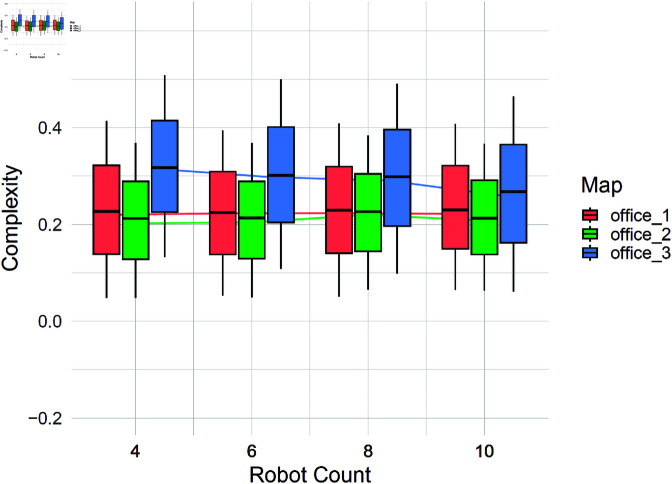
Effect of robot count on computed complexities for the office floor plans. While increasing the number of robots does not reduce complexity for offices 1 and 2, a decreasing trend is observed for office 3

The proposed complexity criterion considers the effect of robot count in countering the complexity of the environment. Fig 10 presents the computed complexities for the office floor plans as a function of robots team size. The results show that for offices 1 and 2, increasing the robot count does not lead to a reduction in computed complexities, suggesting that additional robots do not significantly alter the challenge posed by these layouts. In contrast, for Office 3, an increase in the robot count results in a noticeable decrease in computed complexity, indicating that the added robots contribute to overcoming the structural constraints of the environment.

The accuracy of the current results is limited by the use of 300 simulations per map. While this sample size is substantial enough to generate reasonably accurate point estimates and construct a reliable density estimate, increasing the simulation count would contribute to a more robust understanding of the complexities associated with each environment. This adjustment could lead to more nuanced and reliable insights into the comparative analysis of environments, capturing a broader range of potential scenarios and improving the overall confidence in the obtained results.

## Conclusion

The proposed method introduces a novel criterion for assessing environmental complexity in multi-robot coverage tasks, addressing two key aspects that distinguish it from existing approaches. First, it specifically considers the coverage problem, where robots are required to traverse the entire area, inherently increasing the likelihood of encountering obstacles. Second, it accounts for the effect of swarm size, realizing that a larger team can counter complexity by facing obstacles in parallel, reducing the overall challenge posed by the environment.

The results demonstrate that the proposed measure effectively captures complexity variations across different environments, providing insights into both on-average comparisons, including point estimates and overlapping confidence intervals, and comparing for few numbers of runs, including distributions overlapping ratios.

Future work will involve testing this criterion on additional applications, such as swarm robotic search in complex unknown environments. Additionally, the impact of simplified algorithms, such as GCA, on the results will be investigated.
